# Functional Outcome of Single-Bundle Arthroscopic Anterior Cruciate Ligament Reconstruction using Peroneus Longus Graft and Hamstring Graft: An Open-Label, Randomized, Comparative Study

**DOI:** 10.7759/cureus.60239

**Published:** 2024-05-13

**Authors:** Samir Dwidmuthe, Mainak Roy, Suhas Aradhya Bhikshavarthi Math, Saurabh Sah, Prashant Bhavani, Amey Sadar

**Affiliations:** 1 Orthopaedics, All India Institute of Medical Sciences, Nagpur, Nagpur, IND

**Keywords:** arthroscopy, foot and ankle, peroneus longus graft, hamstring graft, anterior cruciate ligament reconstruction

## Abstract

Background

This study investigates the functional outcomes of single-bundle arthroscopic anterior cruciate ligament (ACL) reconstruction, comparing the use of two distinct graft sources: peroneus longus (PL) graft and hamstring graft. The choice of graft material in ACL reconstruction is crucial for optimal postoperative results, and this study aims to contribute valuable insights into the comparative efficacy of these two graft types.

Method

This open-label randomized comparative study involved a carefully selected cohort of patients undergoing single-bundle arthroscopic ACL reconstruction. Participants were randomly assigned to either the PL graft group or the hamstring graft group. Surgical procedures were conducted using standardized techniques, and postoperative rehabilitation protocols were closely monitored. Functional outcomes, including range of motion, stability, and patient-reported measures, were assessed at predefined intervals to ensure comprehensive data collection.

Results

The study underscores significant demographic and clinical factors in ACL reconstruction outcomes. Participants were predominantly aged 17-30 years (58.33%) with a mean age of 29.27 years and exhibited a male predominance (80.56%). Common complaints included knee pain and instability, primarily due to falls from bikes (55.56%) or sports-related trauma (44.44%). Notably, PL grafts demonstrated advantages over hamstring grafts, with longer mean length (10.11 mm vs. 8.77 mm, p=0.0001) and shorter operation times. Visual analog scale (VAS), International Knee Documentation Committee (IKDC), and Tegner Lysholm scores show no significant differences between grafts over the period of time. There is no notable foot eversion weakness or significant donor site morbidity after the PL graft harvest. Hamstring graft cases exhibit a higher incidence of altered sensation and muscle atrophy, suggesting the potential benefits of PL grafts for improved surgical outcomes.

Conclusions

Graft comparisons favored PL grafts due to longer length, and functional outcome assessments between the two graft types. However, foot and ankle strength assessments revealed fluctuations in strength recovery with PL grafts, highlighting the need for tailored rehabilitation. Thigh circumference variations suggested potential muscle atrophy in the hamstring graft group, along with reported paresthesia in the ipsilateral proximal leg. In conclusion, PL grafts offer potential advantages for ACL surgery, but ongoing monitoring and specialized rehabilitation are crucial.

## Introduction

The knee relies on a complex ligament network, particularly the anterior cruciate ligament (ACL), to stabilize its movement [1]. ACL injuries are a significant concern, especially among athletes, and understanding their epidemiology is essential. Globally, around 200,000 ACL injuries occur annually in the United States alone, with varying rates among athletes in different countries [2]. Non-contact ACL injuries often occur during rapid changes in movement direction, and understanding the specific mechanisms is crucial for prevention [2].

Studies in India highlight soccer as a sport most associated with ACL injuries, often due to non-contact incidents [3]. Factors such as road traffic accidents and twisting injuries contribute significantly to ACL injuries in rural India, affecting more men aged 16-25 years [4].

The ACL is highly susceptible to injury, necessitating effective reconstruction for proper knee joint function. ACL reconstruction (ACLR), a prevalent procedure to restore functional knee stability, necessitates careful consideration of graft selection, a crucial aspect of successful reconstruction [2]. Bone-patellar tendon-bone graft (BPTB) offers advantages like early mobilization and exhibits promising long-term results. However, its utilization presents potential complications at the graft harvesting site, including issues such as patellofemoral pain, quadriceps weakness, donor site morbidity and even patellar fracture [5].

To mitigate these concerns, orthopedic surgeons have explored alternative graft sources, such as the peroneus longus (PL) tendon. PL tendon autograft has gained traction in orthopedic procedures, including deltoid ligament reconstruction and medial patellofemoral ligament (MPFL) reconstruction. The PL tendon possesses a synergistic function with the peroneus brevis tendon, enhancing its appeal as a potential graft source for ACLR [6].

Previous studies have yielded varying insights into the use of the PL tendon as an autograft in ACLR [7,8]. In the pursuit of an ideal autograft, the PL tendon has emerged as a promising option. Its use in ACLR is gaining attention due to its excellent biomechanical properties and high load-to-failure strength. Notably, PL tendon harvest avoids postoperative hamstring muscle weakness and saphenous nerve injury [8]. Moreover, the peroneus brevis tendon, known for its strong ankle eversion function, justifies PL tendon usage [6]. PL tendon has been successfully employed in various ligament and cruciate ligament reconstructions, especially in multi-ligamentous injuries [6].

Other common autograft sources include hamstring tendons (semitendinosus and gracilis (STG)), BPTB, and quadriceps tendons. Recent studies have favored BPTB due to its bone-to-bone healing property, allowing a swift return to professional activities, particularly crucial for athletes. However, BPTB poses risks such as patellar fracture, fat pad fibrosis, and tendon contracture, making hamstring tendons a preferred alternative [5]. Hamstring tendons are easier to harvest with minimal donor site issues and comparable tensile strength to native ACL. Despite these advantages, concerns regarding unpredictable graft size, potential nerve paresthesia, and reduced hamstring muscle strength persist [9].

In this prospective comparative study, we evaluated the functional outcome of single-bundle arthroscopic ACLR using PL tendon and hamstring tendon autografts. The objective of this study was to assess and compare the functional outcomes, donor site morbidity, and improvement in thigh wasting associated with these two autograft options. The findings of this study are expected to significantly contribute to the ongoing advancements in ACLR procedures, enhancing the quality of care for ACL-injured patients.

## Materials and methods

This was a randomized open-label prospective study conducted from April 2022 to December 2023, conducted at All India Institute of Medical Sciences, Nagpur, India. The study was approved by the Institutional Ethical Committee of All India Institute of Medical Sciences, Nagpur (approval number: IEC/Pharmac/2022/385) and registered in the Clinical Trials Registry-India (Registration number: CTRI/2022/06/043039). 

The primary objective of the study was to compare the short-term functional outcomes of single-bundle ACLR using PL graft and hamstring graft. The secondary objectives included evaluating donor site morbidity post PL tendon harvest, assessing changes in thigh circumference, comparing surgical times between graft groups, and checking for sensory loss at the hamstring graft harvest site. The study population consisted of patients aged 18-45 years seeking knee injury treatment at our hospital's Orthopedics Outpatient Department or Emergency Department, diagnosed with complete ACL rupture via clinical examination and MRI. Exclusion criteria were associated grade 4 hondral injuries (>1 cm), meniscal injuries requiring repair, knee arthritic changes, knee fractures, history of prior knee surgery, pre-existing knee deformities, partial ACL ruptures, or foot/ankle deformities precluding PL tendon harvest.

In this study, a sample size of 36 patients was evenly allocated, with 18 participants in each group. Computer-generated random numbers were used for randomization by one investigator. The randomization sequence was securely stored and remained undisclosed to the personnel enrolling patients and those assessing the outcomes. The study was open-label, with participants, surgeons, and investigators aware of the intervention received.

**Figure 1:**
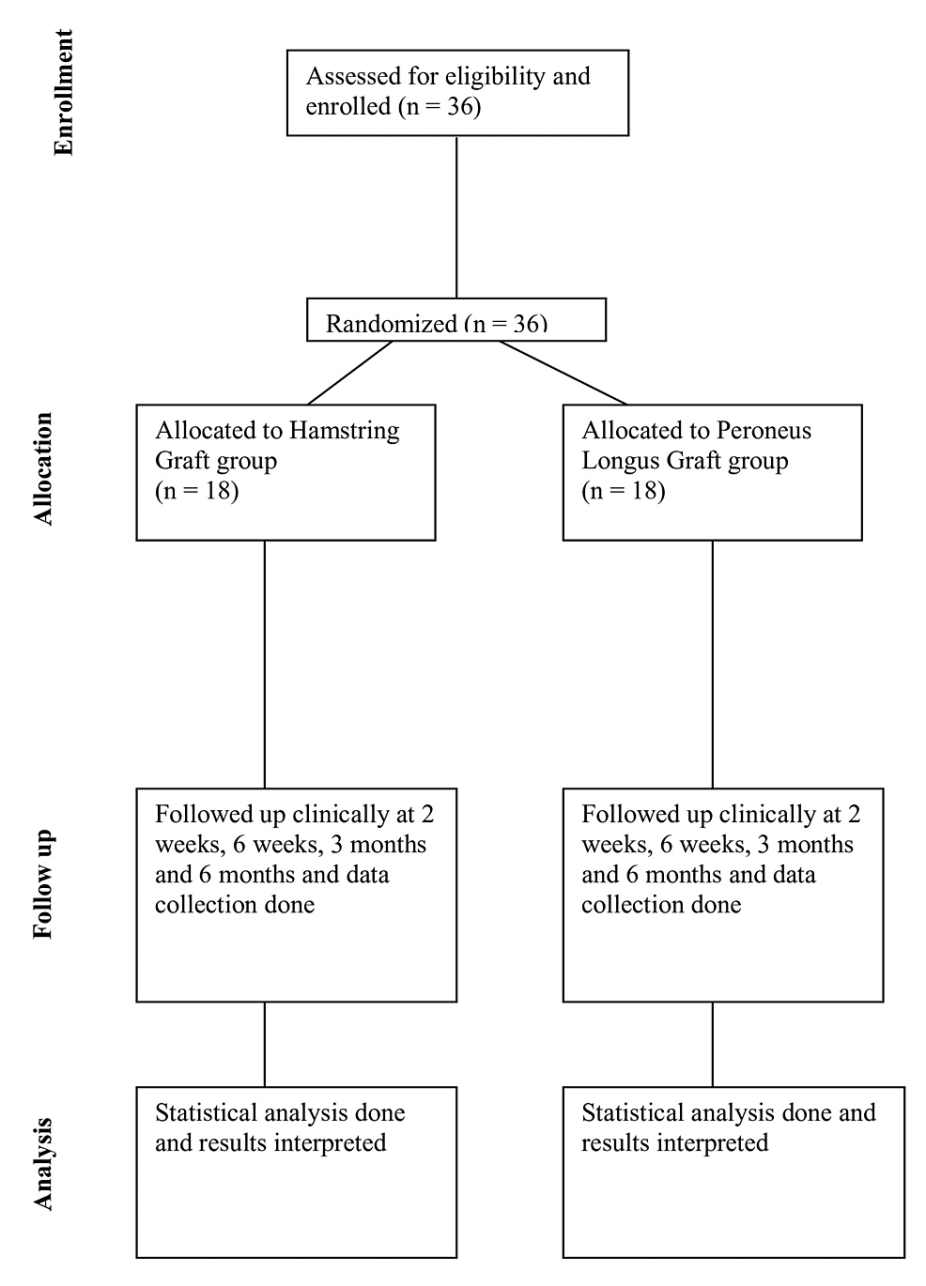
CONSORT chart CONSORT: Consolidated Standards of Reporting Trials

The CONSORT chart for the study is given in Figure 1.

Surgical technique

Standard anteromedial and anterolateral arthroscopic portals were established. Diagnostic arthroscopy was conducted to assess the knee's internal structures. Upon confirming a complete ACL rupture, graft harvesting was carried out based on the group assignment.

Hamstring Graft Harvest

**Figure 2:**
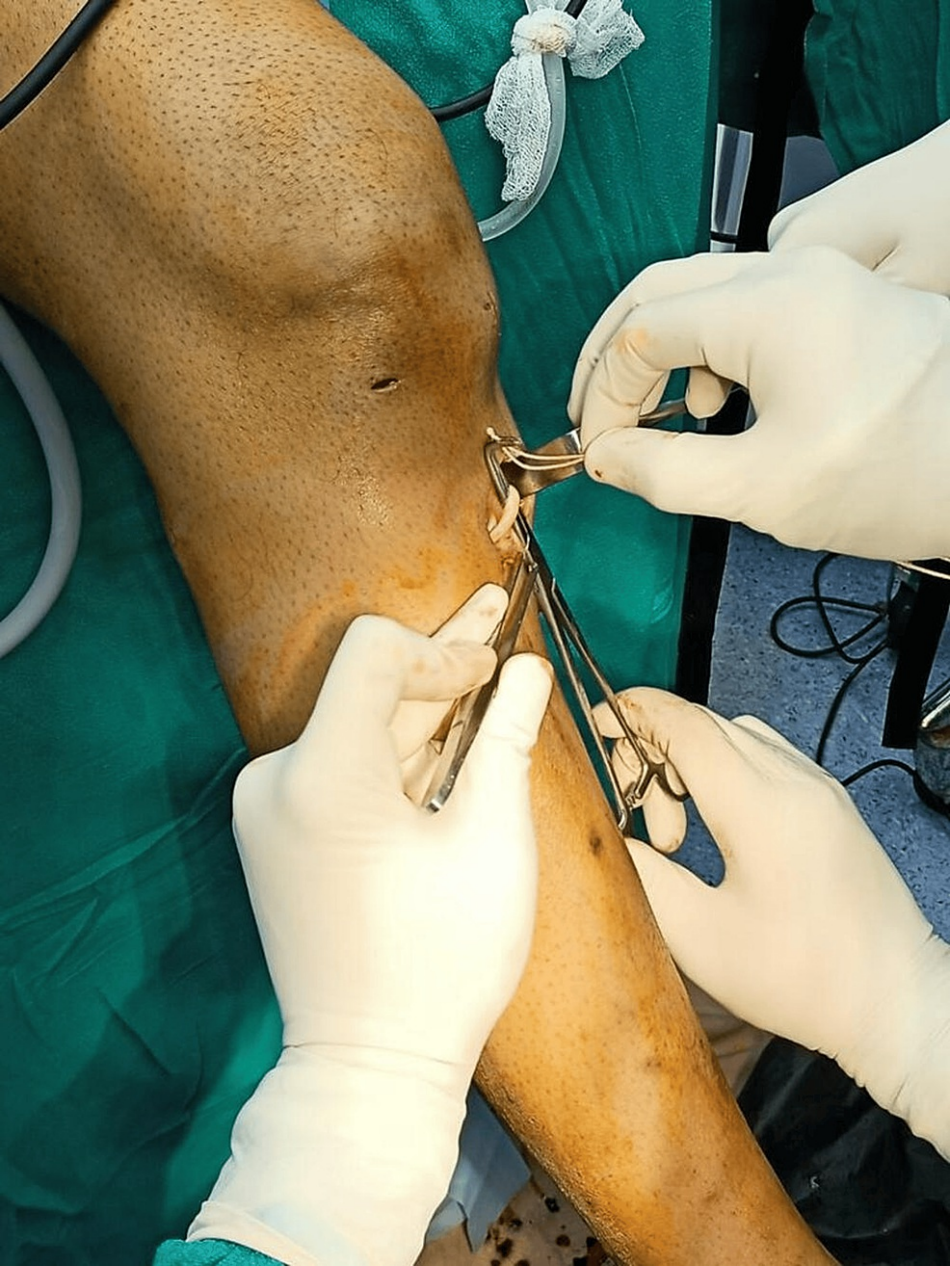
Hamstring tendon exposure

Firstly, a 3 cm longitudinal incision was made at the level of the tibial tuberosity, precisely positioned between the tuberosity and the posteromedial border of the tibia to access the STG tendons. Following this, an oblique incision was made over the sartorius fascia to expose the underlying STG tendons (Figure 2), which were crucial for graft harvesting.

**Figure 3:**
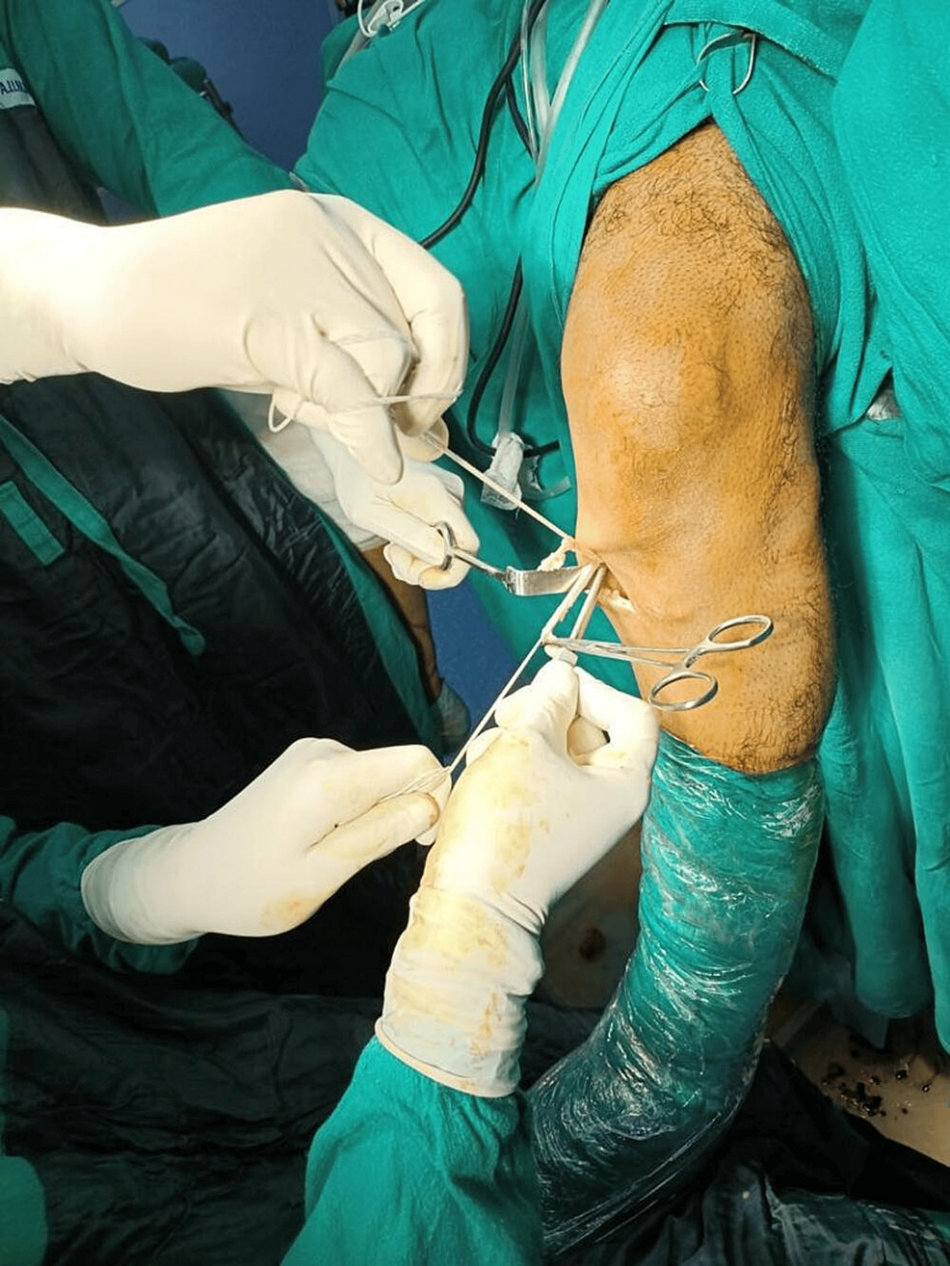
Hamstring tendon harvest by tendon stripper

Once exposed, the STG tendons were harvested using a specialized open tendon stripper (Figure 3), allowing for precise and minimally invasive extraction. Next, attached muscle fibers were meticulously removed from the harvested tendons to ensure the graft consisted solely of the tendon tissue, thereby improving its suitability for ACLR. Finally, depending on the graft length, either a four-strand or five-strand graft was prepared using high-strength polyester sutures to enhance graft strength and durability.

PL Graft Harvest

**Figure 4:**
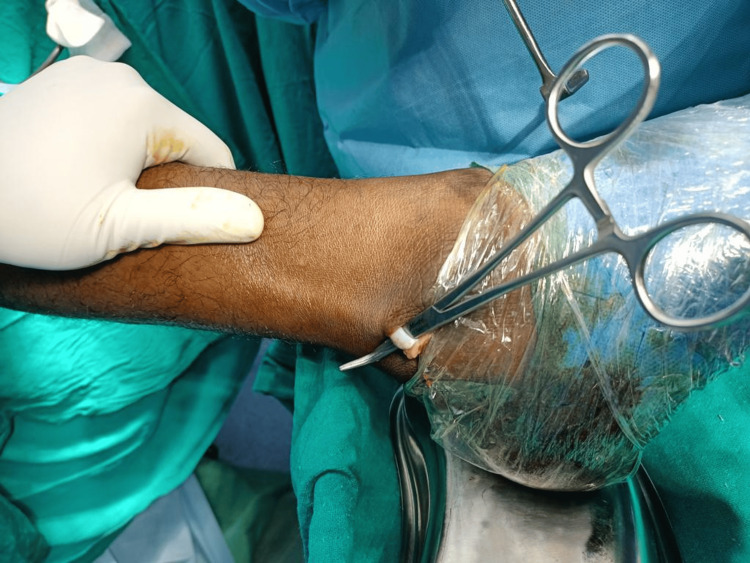
Peroneus longus tendon exposure

Initially, an incision was meticulously planned 3 cm proximal to the most distal point of the lateral malleolus, progressing in a distal-to-proximal direction, penetrating through the skin and subcutaneous tissue. Subsequently, the subcutaneous tissue was dissected to reveal the underlying anatomical structures. Using hemostatic forceps, such as mosquito or Kelly forceps, the PL tendon was identified (Figure 4), with careful attention paid to distinguishing it from the adjacent peroneus brevis tendon.

**Figure 5:**
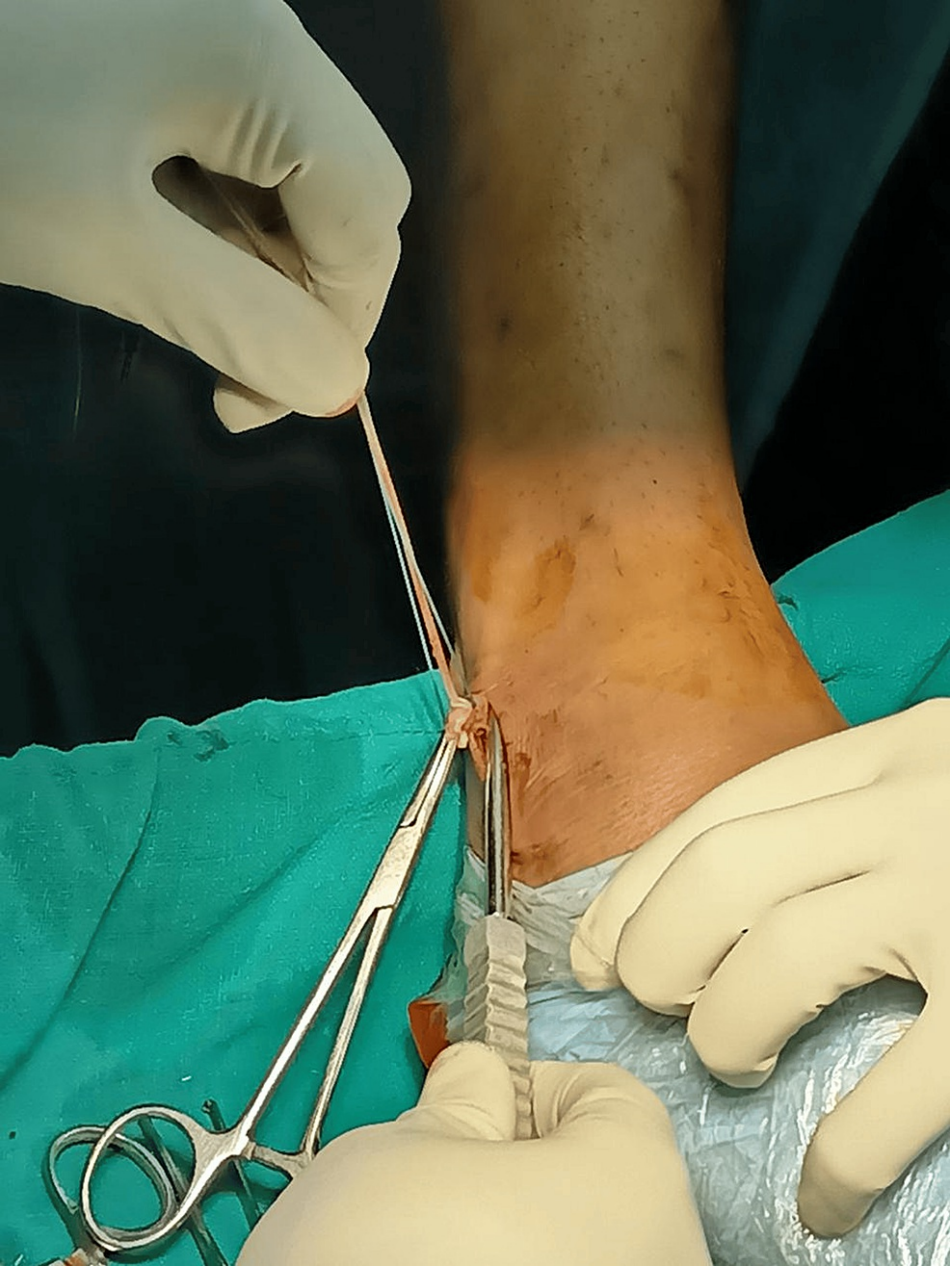
Peroneus longus tendon harvest by tendon stripper

Once isolated, the PL and peroneus brevis tendons were converged and sutured together at the distal end of the incision using No. 1 absorbable sutures to form a unified structure. Proceeding proximally, the PL tendon was incised approximately 5 cm from the fibular head, with extreme care taken to avoid damaging the nearby fibular nerve (Figure 5).

Following this, meticulous dissection was conducted to remove any attached muscle fibers from the PL tendon, ensuring that the harvested graft comprised solely tendon tissue. Finally, the harvested tendon was prepared to create either a two-strand or three-strand graft, selected based on its length, and high-strength polyester sutures were utilized to prepare the graft, ensuring its integrity and durability.

**Figure 6:**
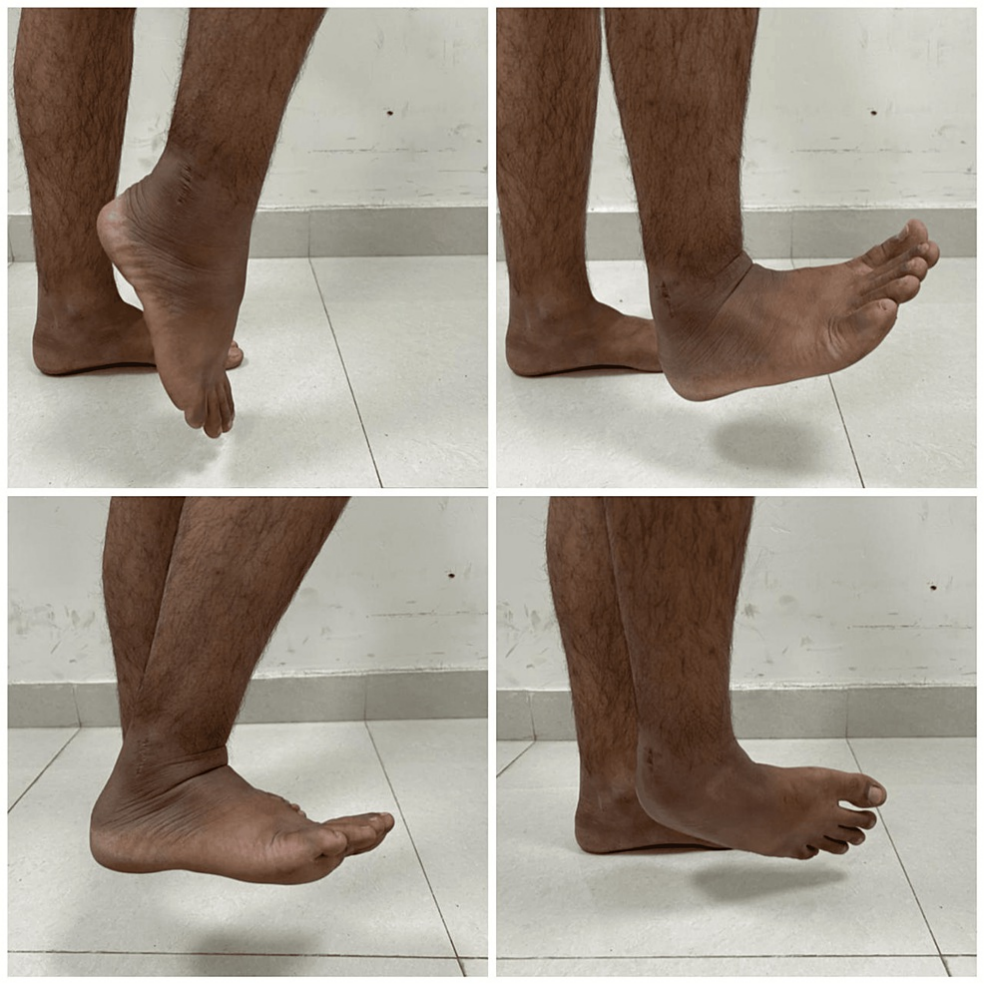
Six-month follow-up of a patient from the PL group showing normal ankle range of motions

**Figure 7:**
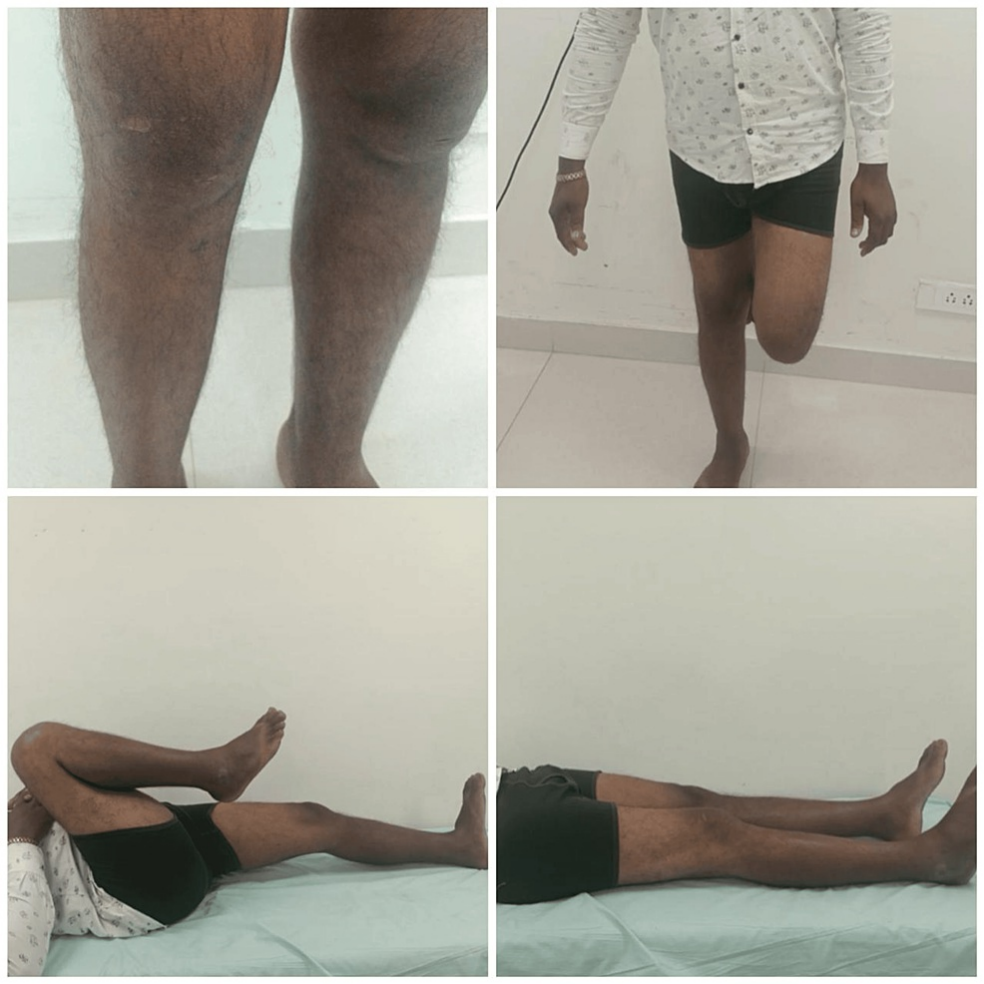
Six-month follow-up of a patient from the Hamstring group showing normal knee range of motions

Any associated meniscal injuries were then evaluated, and partial meniscectomy (removal of damaged portion) was performed. The surgical procedure began with the creation of precise tunnels in the femur and tibia using anatomical landmarks as reference points to accommodate the graft, crucial for graft fixation. Subsequently, the graft, whether hamstring or PL, was passed through the tibial tunnel and anchored on the femoral side using an Endobutton with a closed loop mechanism, ensuring stable fixation. Tibial fixation was achieved with a bio-absorbable interference screw while maintaining the knee flexed at 10 degrees and applying a posterior drawer force to ensure proper tension and stability. In the postoperative phase, intravenous antibiotics were administered for one day to prevent infection, with wound monitoring on the second day. Rehabilitation started on the first day post surgery with a standardized protocol for optimal recovery. Regular follow-up appointments until six months for both groups (Figure 6 and Figure 7) at specific intervals assessed knee stability and function using clinical assessments, alongside standardized measures like the IKDC and Tegner-Lysholm scores. Pain levels were monitored using the VAS, and thigh circumference was measured. Donor-site morbidity in the PL group was assessed using the Foot and Ankle Disability Index (FADI) score, while ankle function was evaluated. 

Statistical analysis of the collected data was performed using IBM SPSS Statistics for Windows, Version 29.0 (Released 2023; IBM Corp., Armonk, New York, United States), utilizing tests such as the Student's t-test to evaluate differences in mean functionality scores at the six-month follow-up post-surgery between the hamstring and peroneus graft source groups. 

## Results

Among the participants, the majority (n=21; 58.33%) fell between the ages of 17 and 30 years, with a mean age of 29.27 years and a standard deviation of 7.87, indicating variability around the mean. Most participants (n=29; 80.56%) identified as male, while a smaller proportion (n=7; 19.44%) were female. Chief complaints included left knee pain and instability in 16 patients (44.44%) and right knee pain and instability in 20 patients (55.56%). The mean duration from injury to surgery was 12.36 months, with a standard deviation of 23.88, highlighting variability in intervention timing. ACL injuries were primarily attributed to falling from a bike in 20 patients (55.56%) and sports-related trauma in 16 patients (44.44%).

Arthroscopic ACLR was performed in 20 patients (55.56%), while the remaining 16 patients (44.44%) underwent a combination of ACLR and operative partial meniscectomy. Hamstring grafts had a mean length of 8.77 mm, whereas PL grafts were longer, with a mean length of 10.11mm, showing a statistically significant difference (p-value = 0.0001). However, there was no statistically significant difference in graft thickness between hamstring and PL grafts. The mean operation time for PL grafts (69.48 minutes) was significantly shorter than for hamstring grafts (84.80 minutes), indicating a notable difference in operation time (p-value = 0.00001). 

The VAS scores for both graft groups decreased over time, indicating a reduction in pain levels. Disparities in VAS scores between the two graft groups were not statistically significant (Table 1). The IKDC scores for both graft groups fluctuated, suggesting potential variations in functional recovery. Discrepancies in IKDC scores between the two graft groups were not statistically significant (Table 2).

**Table 1:** Comparison of VAS scores between hamstring and peroneus longus grafts VAS: Visual Analog Scale

VAS Score	Hamstring Group, mean ± SD	Peroneus Longus Group, mean ± SD	P-Value
Preoperative	4.11 ± 1.99	3.55 ± 2.06	0.424
2 Weeks	7.22 ± 0.94	6.94 ± 1.10	0.424
6 Weeks	4.11 ± 1.18	4.44 ± 1.24	0.4164
3 Months	2.11 ± 1.02	2.44 ± 1.19	0.3758
6 Months	0.88 ± 0.67	0.66 ± 0.59	0.3024

**Table 2:** Comparisons of IKDC scores between hamstring and peroneus longus grafts IKDC: International Knee Documentation Committee

IKDC Score	Hamstring Group, mean ± SD	Peroneus Longus, mean ± SD	P-Value
Preoperative	52.35 ± 11.81	57.27 ± 9.51	0.1779
2 Weeks	39.35 ± 9.74	41.94 ± 14.16	0.5267
6 Weeks	61.29 ± 9.20	61.42 ± 11.59	0.9708
3 Months	78.34 ± 6.79	76.68 ± 9.39	0.5476
6 Months	89.20 ± 4.00	90.8 ± 2.70	0.17

The p-values linked with the Tegner-Lysholm score comparisons consistently suggested that the variances between the hamstring and PL graft groups were not statistically significant (Table 3). Patients with PL grafts demonstrated an improvement in foot and ankle function over time, with FADI scores increasing from 75.70 ± 7.70 at two weeks to 98.93 ± 3.37 at six months. The p-values associated with the score comparisons indicated that the differences in FADI scores between the two graft groups were statistically significant (p < 0.001) (Table 4).

**Table 3:** Comparisons of Tegner-Lysholm scores between hamstring and peroneus longus grafts

Tegner-Lysholm Score	Hamstring Group, mean ± SD	Peroneus Longus, mean ± SD	P-Value
Preoperative	65.38 ± 12.08	66.66 ± 8.67	0.7178
2 Weeks	64.66 ± 11.32	65.61 ± 10.77	0.7992
6 Weeks	83.61 ± 4.71	84.44 ± 3.72	0.5605
3 Months	91.38 ± 2.59	91.5 ± 5.05	0.9344
6 Months	97.38 ± 2.93	97.55 ± 2.97	0.8666

**Table 4:** Comparison of FADI scores between hamstring and peroneus longus grafts FADI: Foot and Ankle Disability Index

FADI Score	Hamstring Group, mean ± SD	Peroneus Longus, mean ± SD	P-Value
Preoperative	100	100	-
2 Weeks	100	75.70 ± 7.70	0.0001
6 Weeks	100	78.06 ± 6.28	0.0001
3 Months	100	88.64 ± 7.26	0.0001
6 Months	100	98.93 ± 3.37	0.1885

At subsequent postoperative time points, the hamstring graft group maintained consistent strength levels of 5/5 in plantar flexion and foot eversion. However, in the PL graft group, the number of participants achieving these strength levels fluctuated over time, with two patients experiencing persistent weakness of 4/5 at six months (Table 5). Additionally, among the cases that used hamstring grafts, five individuals (27.78% of the cases in that group) reported altered sensation in the area where the graft was harvested. Conversely, in the group that utilized PL grafts, none had a similar complication. At various time points, the group with hamstring grafts exhibited a reduction in thigh circumferences compared to the group with PL grafts (Table 6), indicating potential muscle atrophy in the hamstring group.

**Table 5:** Comparison of plantar flexion and foot eversion strength between the hamstring and peroneus longus grafts

Plantar Flexion and Foot Eversion	Hamstring Group, n (%)			Peroneus Longus Group, n (%)		
	3/5	4/5	5/5	3/5	4/5	5/5
Preoperative	_	_	18	_	_	18
2 Weeks	_	_	18	2	12	4
6 Weeks	_	_	18	_	13	5
3 Months	_	_	18	_	7	11
6 Months	_	_	18	_	2	16

**Table 6:** Postoperative differences in thigh circumference between hamstring and peroneus longus grafts

Differences in Thigh Circumference	Hamstring Group, mean	Peroneus Longus Group, mean	P-Value
Preoperative - 2 Weeks	1.36	0.25	0.00001
Preoperative - 6 Weeks	2.17	0.36	0.00001
Preoperative - 3 Months	3.56	0.47	0.00001
Preoperative - 6 Months	3.69	0.5	0.00001

## Discussion

ACL injuries are common and can greatly affect knee function and stability [1]. ACLR surgery is crucial for restoring stability and function [2]. In recent years, different graft options have been explored to improve outcomes [5]. The PL graft, an alternative to the hamstring graft, has emerged as a promising option due to its potential benefits, such as decreased donor site morbidity and similar functional outcomes compared to traditional grafts [7].

In previous studies by Agarwal et al. [10], Keyhani et al. [7], and Rhatomy et al. [11], the mean age of participants ranged from 26.70 to 27.75 years, aligning closely with the current study's age distribution. Our study observed that the majority (58.33%) fell within the 17-30 years age group, with a mean age of 29.27 years and a standard deviation of 7.87. Regarding gender distribution, previous research [7,10,11] showed a predominance of males, ranging from 64.43% to 91.54%. Similarly, in our study, males comprised the majority (80.56%) of participants, while females accounted for a smaller proportion (19.44%), consistent with the higher prevalence of ACL injuries among males.

In the study by Agarwal et al., various modes of ACL injuries were examined [10]. Contact sports injuries accounted for 10.82% of cases, with sportspersons, students, and individuals from other occupations being affected. Non-contact sports injuries comprised 9.79% of cases, mainly involving sportspersons. Road traffic accidents were a prominent cause, representing 32.99% of cases, predominantly affecting individuals from various occupational categories. These findings underscore the significant role of contact sports and road traffic accidents in ACL injuries, highlighting the need for targeted preventive measures. Rhatomy et al. reported that 69% of injuries were sports-related, emphasizing the importance of sports activities in ACL injuries [11]. Additionally, motorcycle injuries constituted 8% of cases, indicating the relevance of vehicular accidents. In the current study, 55.56% of ACL injuries resulted from bike falls, while 44.44% were sports-related traumas.

Analyzing various studies on ACLR, particularly focusing on graft diameter, reveals important clinical insights. Keyhani et al. observed a significant difference in graft diameter between PL tendon and hamstring autografts, with the former having a larger diameter [7]. This finding was consistent with our study, where the mean diameter for the PL group was 10.11 mm compared to 8.77 mm for the hamstring group.

Studies by Magnussen et al. [12] and Conte et al. [9] emphasized the heightened risk associated with grafts smaller than 8 mm, underlining the importance of graft stability in preventing post-ACLR re-ruptures. Consequently, in clinical decision-making, graft diameter emerges as a crucial factor in graft selection. Surgeons should prioritize grafts with a diameter of at least 8 mm to enhance stability and mitigate the risk of re-rupture.

The Lysholm Knee Score is a questionnaire utilized to evaluate functional disability in patients with knee disorders following ACLR, with scores ranging from 0 to 100, categorized as excellent, good, fair, or bad based on specific score ranges. Studies by Agarwal et al. [10], Keyhani et al. [7], and Rhatomy et al. [11] comprehensively analyzed knee function and compared outcomes between different surgical groups, particularly focusing on the PL graft group and the hamstring tendon graft group. Statistical analyses revealed no significant differences in Lysholm knee scores between these groups, indicating comparable functional recovery outcomes. Additionally, studies by Kerimoglu et al. [8] and He et al. [13] demonstrated positive results and better Lysholm knee scores with ACLR using the PL tendon graft, further affirming its effectiveness in improving knee function post surgery. Our study showed comparable results in this score between the two groups in successive follow-ups.

The IKDC score is a crucial tool for assessing knee function, symptoms, and engagement in activities and sports, with scores ranging from 0 to 100. Agarwal et al.'s study found no statistically significant differences in IKDC scores between the two graft groups, indicating comparable functional outcomes [10]. Similarly, a systematic review by He et al. also supported comparable functional outcomes in IKDC scores between patients with PL tendon autograft ACLR and those with hamstring tendon grafts [13]. The mean IKDC scores in the present study further reinforce the lack of significant differences between the hamstring and PL graft groups, consistent with previous findings.

Agarwal et al.'s findings revealed that the PL graft group exhibited no thigh muscle atrophy as compared to the hamstring tendon graft group, emphasizing the potential advantage of the PL graft [10]. Consistent results were observed by Rhatomy et al., affirming that the PL graft group displayed significantly less thigh hypotrophy and better postoperative recovery compared to the hamstring tendon graft group [11]. Thigh hypotrophy due to hamstring tendon harvest was highlighted as a concern as it lead to long-term deranged knee kinematics in an already injured knee, underlining the importance of selecting an appropriate graft type to mitigate such issues. Keyhani et al. further reinforced the superiority of the PL graft in minimizing thigh hypotrophy [7]. Their study demonstrated considerably less thigh hypotrophy in the PL group compared to the hamstring tendon group, supporting the notion that the choice of graft significantly influences muscle recovery and thigh circumference. Our study also suggests that the hamstring graft group was associated with thigh atrophy while it was absent in the PL tendon graft group.

The FADI score is a key measure for assessing foot and ankle function following ACLR. Angthong et al. highlighted potential complications such as reduced peak torque in ankle eversion and inversion, as well as decreased ankle function and stability following PL tendon harvesting [14]. Keyhani et al.'s study revealed no significant difference between the donor and healthy contra lateral side FADI score, indicating no donor site discomfort after PL tendon harvest [7]. In our study, both hamstring and PL graft groups displayed optimal foot and ankle function preoperatively, with perfect FADI scores of 100. Subsequent postoperative assessments showed improvement in foot and ankle function for the PL graft group, with increasing FADI scores over time from 75.70 ± 7.70 at two weeks to 98.93 ± 3.37 at six months. Agarwal et al. further emphasized the lack of patient complaints regarding foot eversion following PL tendon harvesting, attributing it to the dominance of the peroneus brevis in ankle eversion and the regenerative potential of the harvested graft [10]. Their study underscores the safety of PL tendon harvesting concerning ankle morbidity. In our study, the PL graft group in the immediate postoperative period had mild to moderate weakness in foot eversion (mostly owing to pain from graft harvest) but later displayed notably fair foot eversion strength at the six-month follow-up with a proper rehabilitation program. However, two patients had residual weakness at a level of 4/5 after six months. All of this demonstrates that there is not much significant donor site morbidity post PL graft harvest. In a comparative analysis, Kjaergaard et al. emphasized hypoesthesia as a significant donor site morbidity in hamstring tendon harvesting [15]. In our study, hypoesthesia was reported by 27.78% of patients with hamstring grafts but none with PL grafts, suggesting it is a common complication in the hamstring graft group.

Limitation of the Study

The study had a small sample size of 36 participants (18 in each group) via convenient sampling. However, despite the small sample size, the study aimed to maximize available resources to provide valuable insights into the comparative outcomes of both surgical procedures. 

## Conclusions

This study compared short-term functional outcomes of ACLR using PL graft and hamstring graft in predominantly young adults aged 18-45 years with diverse occupations. Road traffic accidents were identified as a common cause of ACL tears. Graft comparisons favored PL grafts due to longer length, and functional outcome assessments using validated scales showed comparable results between the two graft types. However, foot and ankle strength assessments revealed fluctuations in strength recovery with PL grafts, highlighting the need for tailored rehabilitation. Some patients in the PL group exhibited residual foot eversion weakness at the donor site. Thigh circumference variations suggested potential muscle atrophy in the hamstring graft group, along with reported paresthesia in the ipsilateral proximal leg. In conclusion, PL grafts offer potential advantages for ACL surgery, but ongoing monitoring and specialized rehabilitation are crucial. The study contributes to understanding ACLR methods and graft choices, supporting evidence-based orthopedic practices.
